# Immunosenescence and Vaccine Efficacy in Aging: Dynamic Interplay of Gut Microbiota and mTOR Signaling Pathways

**DOI:** 10.1111/acel.70548

**Published:** 2026-05-22

**Authors:** Jiaxuan Li, Yuhong Zhang, Daijun Yu, Jianhua Li, Keda Chen, Lisheng Chu

**Affiliations:** ^1^ Zhejiang Chinese Medical University Hangzhou China; ^2^ Zhejiang Key Laboratory of Public Health Detection and Pathogenesis Research, Department of Microbiology Zhejiang Provincial Center for Disease Control and Prevention Hangzhou P.R. China; ^3^ Key Laboratory of Artificial Organs and Computational Medicine in Zhejiang Province, Shulan International Medical College Zhejiang Shuren University Hangzhou P.R. China

## Abstract

Aging significantly impairs vaccine efficacy in older adults, driven by immunosenescence, inflammaging, and disruptions in the gut microbiota‐mTOR‐immune axis. This review synthesizes current evidence on how aging alters vaccine‐induced immune responses through the interplay of gut microbiota dysbiosis and dysregulated mTOR signaling. Age‐related microbial diversity declines and reduced short‐chain fatty acid (SCFA) production exacerbate inflammation, while heightened mTOR activity suppresses autophagy, promotes pro‐inflammatory states, and impairs T/B cell function, collectively diminishing antibody production and immune memory formation. We highlight the bidirectional interaction between SCFAs and mTOR, where SCFAs mitigate mTOR overactivation to enhance immune regulation, and mTOR dysregulation further aggravates microbial dysbiosis, forming a vicious cycle. Critically, this review systematically stratifies the evidence, distinguishing preclinical mechanistic insights from correlative human data. Animal and human studies suggest that targeting this axis—via mTOR inhibitors, probiotics, or dietary interventions—holds promise for improving vaccine responses in the elderly. We propose future research directions, including personalized vaccine strategies leveraging microbiota profiling and mTOR modulation, to address the challenges of infection in aging populations and advance precision medicine for healthy aging.

## Graphical abstract

Aging impairs vaccine efficacy through gut microbiota dysbiosis and mTOR hyperactivation, which together drive inflammaging and weaken immune memory. This review highlights the bidirectional microbiota–mTOR axis and proposes that combining mTOR inhibitors with microbiota modulation offers a promising strategy to enhance vaccine responses in older adults.

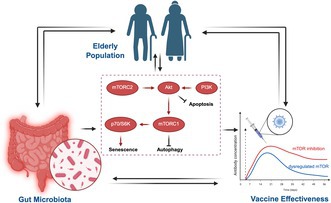

## 1 Introduction

As global population aging accelerates, older adults face heightened susceptibility to infectious diseases due to immunosenescence and comorbidities, placing greater demands on preventive measures such as vaccination. According to United Nations data, by 2050, individuals aged 65 and older are projected to account for more than 16% of the global population, a substantial rise from 9% in 2020. Due to age‐related immune decline and an increased burden of comorbidities, older populations exhibit significantly reduced resistance to infectious diseases such as influenza, pneumococcal infections, and coronavirus disease 2019 (COVID‐19), resulting in higher incidence rates, hospitalization rates, and mortality (Bracken et al. 2025). This not only places a greater strain on public health system resources but also poses a formidable challenge to the effectiveness of vaccines as a primary preventive measure. Clinical studies demonstrate that older adults generally exhibit a diminished immune response to common vaccines compared to younger individuals. For instance, the protective efficacy of influenza vaccines in people aged 65 and older ranges from only 30% to 50%, substantially lower than the 70% to 90% observed in younger populations, underscoring the pronounced suppressive effect of aging on vaccine efficacy (Chen et al. 2022; Jiang et al. 2025).

Extensive evidence indicates that age‐related immune system deterioration, termed immunosenescence, along with its accompanying chronic low‐grade inflammation, known as inflammaging, are critical factors undermining vaccine‐induced immune responses (Liu et al. 2023). Immunosenescence is characterized by reduced T‐cell and B‐cell responsiveness, diminished antigen presentation efficiency, and impaired immune memory formation, while inflammaging exacerbates this process by disrupting immune homeostasis through the persistent accumulation of pro‐inflammatory cytokines, such as interleukin‐6 (IL‐6) and tumor necrosis factor‐alpha (TNF‐α) (Liu et al. 2023). Recent studies have highlighted the pivotal role of the gut microbiota and its metabolites, such as short‐chain fatty acids (SCFAs), in regulating immune homeostasis and vaccine efficacy (Tang et al. 2022). As the largest microbial ecosystem in the human body, the gut microbiota influences both local and systemic immune responses through metabolic products, interactions with immune cells, and maintenance of gut barrier function (Li et al. 2024). However, aging profoundly alters its composition and functionality, manifesting as a decline in microbial diversity, a reduction in beneficial bacteria (e.g., *Bifidobacterium*), and an increase in pathogenic taxa (e.g., *Proteobacteria*). This dysbiotic pattern not only intensifies inflammaging but may also impair vaccine‐induced adaptive immune responses through complex molecular mechanisms, such as reduced antibody titers and T‐cell activity (Kawamoto and Hara 2024; Yang et al. 2025).

As a central hub of cellular metabolism and immune regulation, the mechanistic target of rapamycin (mTOR) signaling pathway serves as a critical bridge in this process (Regan et al. 2022). mTOR activity becomes aberrantly elevated with age, closely correlating with microbial dysbiosis and immune decline (Costa et al. 2025). By suppressing autophagy, promoting inflammation, and modulating T‐ and B‐cell functions, mTOR directly or indirectly influences vaccine responsiveness (Lu et al. 2022). Studies have shown that SCFAs can mitigate inflammation and enhance immune responses by downregulating mTOR activity, whereas the age‐related reduction in SCFA production may amplify mTOR's detrimental effects, creating a vicious cycle (Kawamoto and Hara 2024). This dynamic interplay between mTOR and the gut microbiota suggests that their integrated effects may represent a key mediator of aging's suppressive impact on vaccine efficacy (Xiao et al. 2025). Although existing research has begun to elucidate the independent roles of mTOR and the gut microbiota in immunosenescence—for instance, mTOR inhibitors (e.g., rapamycin) improving vaccine responses in animal models or probiotic interventions boosting antibody production in preclinical studies—the molecular details of their synergistic actions and their specific contributions to vaccine immunity remain underexplored (Liang et al. 2022). This is particularly true in human aging populations, where causal relationships have yet to be fully validated (Ji et al. 2025).

Building on this foundation, this review aims to systematically explore how aging influences vaccine efficacy through the gut microbiota and the mTOR pathway, with a focus on the pathological underpinnings of immunosenescence, inflammaging, and the gut‐immune axis. It will dissect the molecular mechanisms underlying the interplay between mTOR and the microbiota and synthesize their synergistic roles in shaping vaccine responses. By integrating the latest advances from animal models and human studies, we aim to elucidate the dynamic regulatory network of the mTOR‐microbiota‐immune axis in aging and its impact on vaccine‐induced protective efficacy. Furthermore, this article will outline future research directions, such as the synergistic potential of mTOR‐targeted interventions combined with microbiota modulation, as well as the prospects for personalized vaccine strategies tailored to individual microbiota profiles. To ensure scientific rigor, we explicitly contextualize the evidence hierarchy throughout, delineating where conclusions are driven by animal models versus human correlative studies. Ultimately, this review seeks to provide theoretical insights and practical guidance for enhancing immune protection in older adults and addressing the challenges posed by infectious diseases in aging societies.

## 2 Aging and Changes in the Immune System

### 2.1 Immunosenescence and Inflammaging

The impact of aging on the immune system is gradual and complex, with immune function undergoing significant deterioration over time, a process known as immunosenescence. One of the hallmark features of immunosenescence is the weakening of adaptive immunity, particularly the decline in T‐cell responsiveness, which is closely tied to thymic involution. The thymus, a critical organ for T‐cell development and maturation, begins to atrophy after puberty and by old age is almost entirely replaced by adipose tissue, resulting in a drastic reduction in the production of naïve T cells. Studies estimate that in individuals over 70 years of age, thymic output is reduced to approximately 10% of that in young adults, accompanied by a marked decline in T‐cell receptor (TCR) diversity (Arango‐Franco et al. 2024). Reduced naïve T‐cell output due to thymic involution, altered naïve‐to‐memory and CD4‐to‐CD8 ratios, increased exhausted memory T cells, and overall impaired T‐cell proliferative and effector functions. Furthermore, chronic antigenic stimulation and persistent viral infections, such as cytomegalovirus (CMV), exacerbate T‐cell aging. The widespread prevalence of CMV infection in older populations drives excessive differentiation and functional exhaustion of effector T cells, characterized by diminished proliferative capacity and reduced cytokine secretion, limiting the body's ability to respond to new pathogens or vaccine antigens (Dahlquist et al. 2024). Concurrently, B‐cell‐mediated humoral immunity is also significantly compromised. In older adults, the generation of B‐cell precursors in the bone marrow decreases, impairing antibody production, particularly the formation of high‐affinity antibodies and memory B cells (Goronzy and Weyand 2005). Research shows that the affinity maturation of immunoglobulin G (IgG) antibodies post‐vaccination is hindered in older adults, directly undermining long‐term protection against infections and vaccines (Bracken et al. 2025; Qi et al. 2014). While certain aspects of innate immunity show hyperactivation—such as persistent pro‐inflammatory cytokine release by macrophages and dendritic cells—other key functions, including chemotaxis and phagocytosis, are impaired, leading to inefficient pathogen clearance despite chronic inflammation (Liu et al. 2023). This imbalance between innate and adaptive immunity exacerbates overall immune dysregulation, laying the groundwork for heightened susceptibility to infectious diseases and diminished vaccine responses.

**FIGURE 1:**
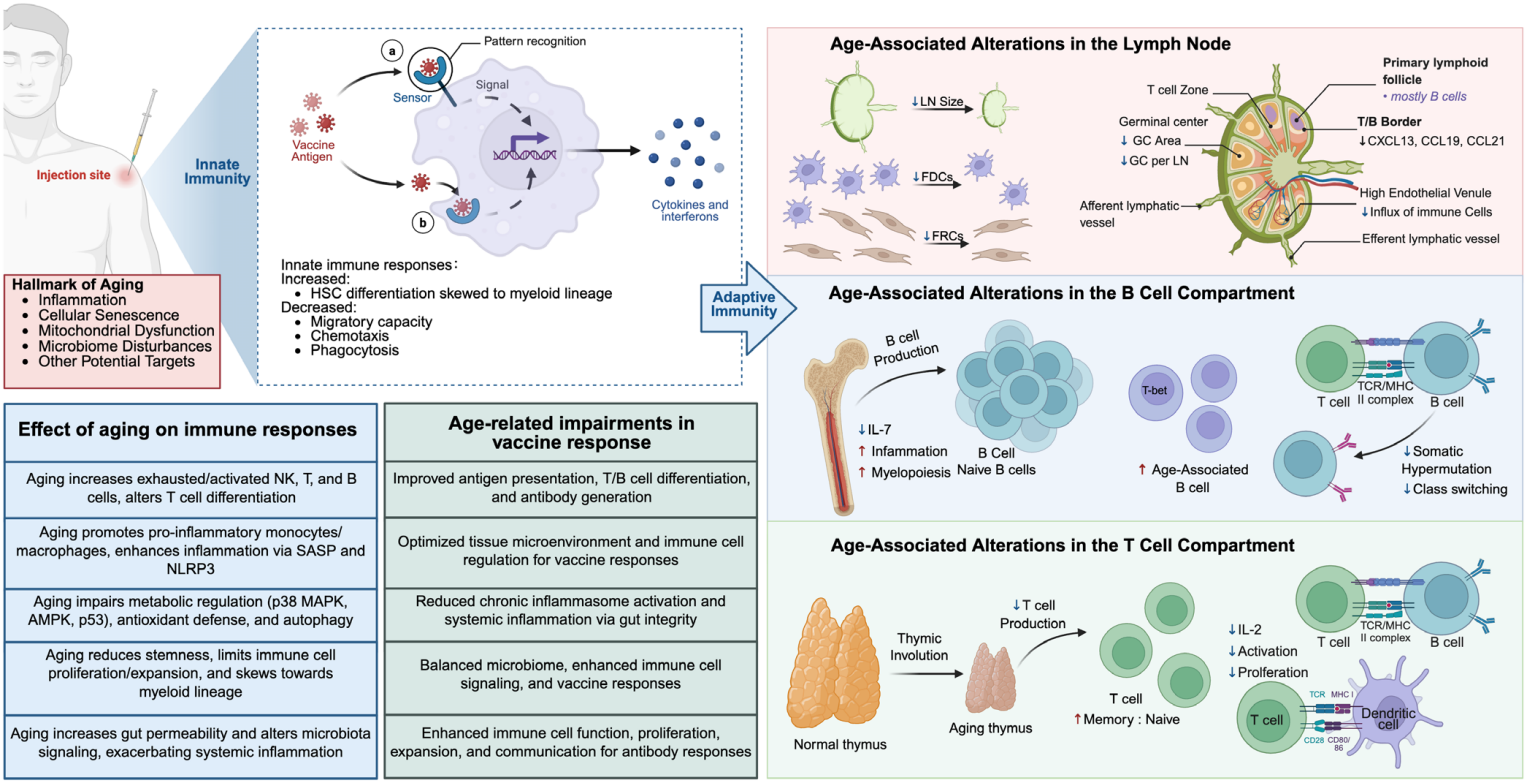
Age‐related alterations in lymphoid tissues and immune responses impacting vaccine efficacy. Vaccination primarily elicits both innate and adaptive immune responses through intramuscular injection, often supplemented with adjuvants to enhance immunogenicity. Following administration, antigens are captured by dendritic cells (DCs), which activate pattern recognition receptors (PRRs) and secrete chemokines to recruit monocytes from circulation. These monocytes migrate to the injection site, acquire antigens, and undergo differentiation. Antigen‐experienced innate immune cells traffic to the draining lymph nodes, where they present antigens via Major Histocompatibility Complexes (MHC) to activate naïve T cells through T cell receptors (TCRs), initiating the adaptive immune response. Aging induces hallmarks such as cellular senescence, inflammation, and mitochondrial dysfunction, skewing innate and adaptive immunity. In lymph nodes, aging reduces size, GC area, and FDC numbers, impairing B‐ and T‐cell interactions. Thymic involution decreases naïve T‐cell output, while increased exhausted T cells and pro‐inflammatory monocytes (via NLRP3) reduce vaccine responses. Dysregulated mTOR and microbiota exacerbate systemic inflammation, impairing antibody production. Potential interventions include optimizing the tissue microenvironment, reducing inflammation, and balancing microbiota to enhance vaccine efficacy in older adults.

Inflammaging, a chronic, low‐grade inflammatory state associated with aging, further accelerates the progression of immunosenescence. Its defining characteristic is the sustained elevation of pro‐inflammatory cytokine levels, including IL‐6, TNF‐α, and other inflammatory markers like C‐reactive protein (CRP) (Zhao et al. 2024). This chronic accumulation stems from multiple sources, such as the senescence‐associated secretory phenotype (SASP) of aging cells, gut microbiota dysbiosis, and the buildup of metabolic waste (Scisciola et al. 2023; Seymour et al. 2022). Senescent cells, which accumulate in aging tissues, secrete SASP components—including pro‐inflammatory cytokines and matrix metalloproteinases—that not only induce local inflammation but also propagate systemic effects via paracrine signaling (Zhang et al. 2024). Additionally, the accumulation of metabolic byproducts, such as oxidized lipids and advanced glycation end‐products (AGEs), further activates innate immune receptors (e.g., Toll‐like receptors, TLRs), amplifying inflammatory signaling (Dong et al. 2025). Inflammaging not only hastens immune cell aging but also reinforces pro‐inflammatory signaling through positive feedback loops, perpetuating a vicious cycle (Aranda et al. 2024). For example, chronically elevated IL‐6 can further stimulate inflammation via the NF‐κB pathway, while TNF‐α may induce apoptosis and tissue damage, contributing to sustained immune deterioration (Ajoolabady et al. 2023; Walker et al. 2022). This persistent inflammatory microenvironment not only weakens pathogen clearance but also significantly disrupts vaccine‐induced immune responses. Studies indicate that elevated IL‐6 levels in the context of inflammaging suppress antibody class‐switching in B cells, reducing the protective efficacy of post‐vaccination antibodies (Olivieri et al. 2023). Collectively, the interplay between immunosenescence and inflammaging markedly diminishes immune defenses in older individuals, providing a pathological basis for the increased incidence of infectious diseases and reduced vaccine effectiveness (Figure 1).

Clinical trials underscore these age‐related disparities in vaccine responses. In a phase 1/2 trial among Chinese adults aged 18 and older, the immunogenicity and safety of the BBIBP‐CorV COVID‐19 vaccine revealed that the geometric mean titer (GMT) of neutralizing antibodies in the ≥ 60 age group was significantly lower than in younger cohorts (Xia et al. 2021). Similarly, a phase I trial of the recombinant COVID‐19 vaccine V‐01 (containing an IFN‐PADRE‐RBD‐Fc dimer fusion protein) in Chinese participants—divided into healthy young adults (18–59 years) and older adults (≥ 60 years)—demonstrated that GMTs for neutralizing antibodies and receptor‐binding domain (RBD) binding capacity were lower in the older group (Zhang et al. 2021). In Russia, a study of Gam‐COVID‐Vac (a prime‐boost vaccine based on rAd26 and rAd5 vectors) reported efficacy rates in healthy individuals as follows: 91.9% (18–30 years), 90% (31–40 years), 91.3% (41–50 years), 92.7% (50–60 years), and 91.8% (> 60 years) (Wu et al. 2021). A randomized trial evaluating the safety and immunogenicity of the inactivated SARS‐CoV‐2 vaccine CoronaVac in Chinese adults aged ≥ 60 showed that neutralizing antibody GMTs were negatively correlated with age, with the ≥ 70 age group exhibiting lower GMTs than the 60–64 and 65–69 age groups across both trial phases (Wu et al. 2021). In the United States, a parallel study of the Moderna mRNA‐1273 SARS‐CoV‐2 vaccine in adults aged ≥ 56 found that, 57 days post‐vaccination, the 56–70 age group had significantly higher neutralizing antibody GMTs than the ≥ 71 age group (Anderson et al. 2020). Comparisons between the 18–55 and ≥ 55 age groups revealed significant differences in vaccine responses at days 29, 43, and 57 post‐vaccination with Moderna mRNA‐1273, with lower neutralizing antibody GMTs detected in the ≥ 55 group (Chu et al. 2021). These clinical disparities not only underscore age‐related decline but also hint at potential platform‐specific efficacies, warranting mechanistic exploration. Similarly, in U.S. cohorts, the efficacy of Moderna mRNA‐1273 (100 μg dose) was 95.6% in those aged 18–65, compared to 86.4% in those ≥ 6529. A UK phase 2/3 trial of the ChAdOx1 nCoV‐19 vaccine in young and older adults showed that anti‐spike IgG responses in the ≥ 70 age group were lower than in the 18–55 and 56–69 age groups, with values of 3565 AU/mL, 6439 AU/mL, and 4553 AU/mL in the low‐dose group, and 4156 AU/mL, 9807 AU/mL, and 5496 AU/mL in the standard‐dose group, respectively (Baden et al. 2021). In contrast, a clinical trial of the Pfizer‐BioNTech BNT162b2 vaccine in the 18–55 and 65–85 age groups observed comparable dose‐dependent SARS‐CoV‐2 neutralizing GMTs across both age groups, suggesting an exception to the typical age‐related decline (Walsh et al. 2022).

### 2.2 The Role of mTOR in Immunesenescence

**FIGURE 2:**
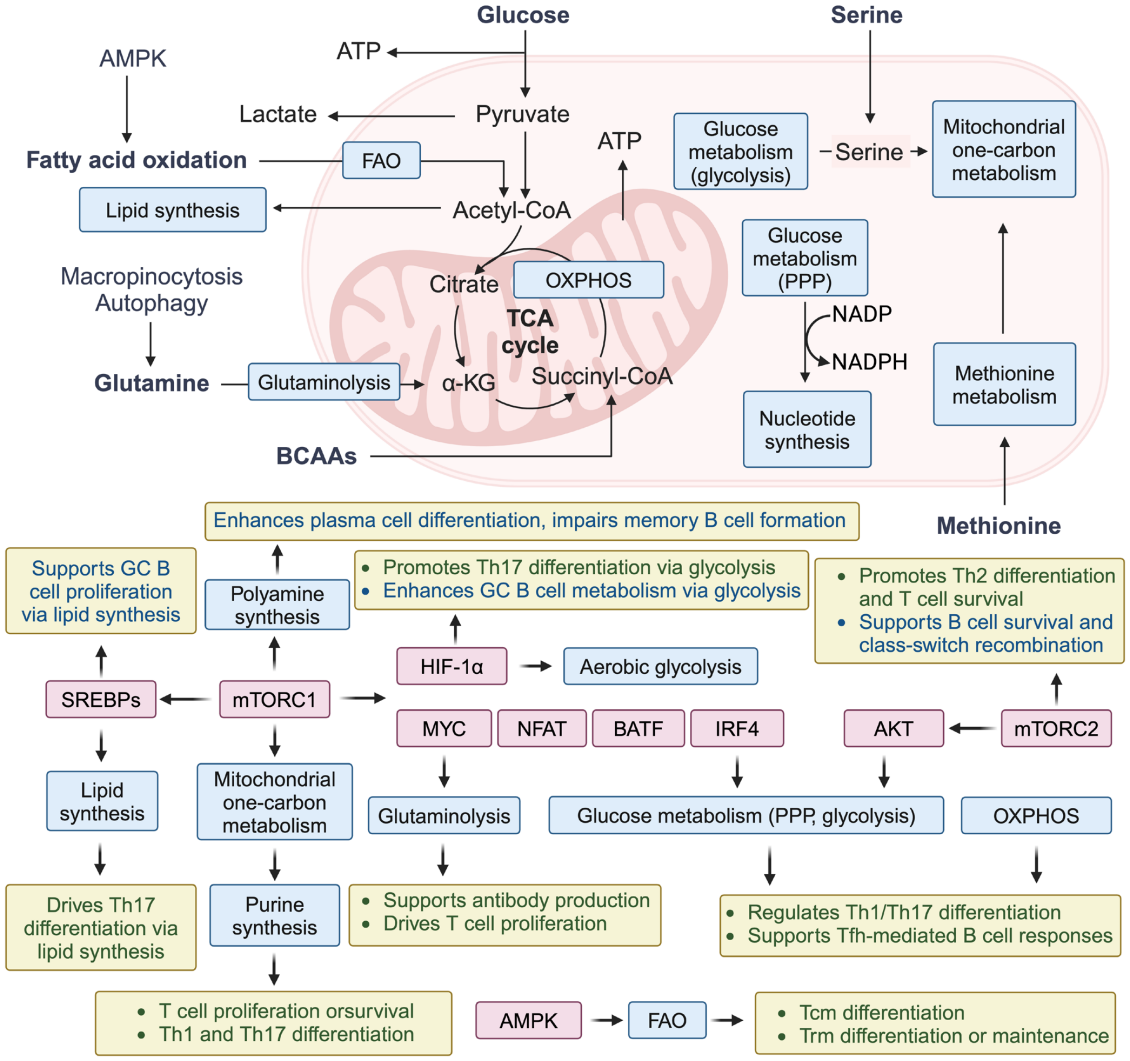
Metabolic pathways regulating immune cell differentiation and function. Immune cell activation and differentiation are tightly regulated by metabolic pathways, including glycolysis, OXPHOS, FAO, and amino acid metabolism. Glucose is metabolized via glycolysis to generate ATP and pyruvate, which feeds into the TCA cycle, fueling OXPHOS and supporting cellular energy demands. FAO and lipid synthesis contribute to memory T cell (Tcm, Trm) differentiation and B cell proliferation. Amino acid metabolism, including glutaminolysis, serine metabolism, and methionine metabolism, influences immune cell fate decisions by supporting nucleotide synthesis, polyamine synthesis, and mitochondrial function. The mTORC1 and HIF‐1α signaling pathways promote aerobic glycolysis, Th17 differentiation, and GC B cell metabolism, while AMPK activation favors FAO and memory T cell maintenance. Methionine metabolism regulates Th2 differentiation and supports class‐switch recombination in B cells. Collectively, these metabolic programs orchestrate immune responses by directing cell fate, function, and survival.

Immunosenescence is a multifaceted process, and its associated immunometabolic regulatory mechanisms have emerged as a focal point in current research. During metabolic adaptation to acute and chronic infections, naïve T cells and B cells exit their quiescent state, rapidly upregulating glucose metabolism, nutrient uptake, and anabolic pathways, accompanied by lactate production. Additionally, mitochondrial metabolism and oxidative phosphorylation (OXPHOS) are enhanced (Chapman et al. 2020; Sadoff et al. 2021). These events are orchestrated by selective transcription factors that induce essential gene expression programs to support anabolic metabolism, including *MYC* (Wang et al. 2011), *IRF4* (Man et al. 2013), *BATF* (Kurachi et al. 2014), *NFAT* (Klein‐Hessling et al. 2017), *SREBPs* (Kidani et al. 2013), and signaling pathways linked to the mechanistic target of rapamycin (mTOR) (Tan et al. 2017). Consequently, inhibiting anabolic metabolism impairs lymphocyte activation and proliferation (Bailis et al. 2019). Effector T‐cell and B‐cell subsets also exhibit distinct metabolic profiles in glucose, polyamine, purine, lipid, and glutamine metabolism, which regulate their differentiation and function (Wagner et al. 2021). These discrete metabolic programs rely on transcription factors capable of sensing metabolic signals, such as hypoxia‐inducible factor 1‐alpha (HIF‐1α) (Clever et al. 2016). Anabolic metabolism drives cellular activation and differentiation; however, metabolic adaptations persist throughout infection, influencing cell fate, function, and localization (Figure 2).

Aberrant activation of the mTOR pathway plays a critical role in immunosenescence. With advancing age, the activity of mTOR complex 1 (mTORC1) increases, and overactivation of its key component, Raptor, drives T‐cell senescence, leading to cell cycle arrest and promoting the secretion of pro‐inflammatory cytokines such as IL‐6 and TNF‐α (Ortega‐Molina et al. 2024). Moreover, in T cells deficient in RIPK1, aberrant mTORC1 activation directly induces mitochondrial dysfunction and oxidative stress (via ROS accumulation), accelerating cellular senescence (Imanishi et al. 2023). mTORC1 also inhibits the clearance of damaged organelles by suppressing the autophagy regulator transcription factor EB (TFEB), resulting in the accumulation of metabolic waste (Lee and Schreiber 2025). In aged T cells, defective mitophagy triggers ROS bursts, further damaging DNA and protein function (Morris et al. 2022). Additionally, excessive mTOR activation alters immune cell function through metabolic reprogramming (e.g., enhanced glycolysis) and epigenetic modifications (e.g., histone acetylation) (Panda et al. 2009). In aging macrophages, mTOR‐dependent activation of the NF‐κB signaling pathway promotes the secretion of TNF‐α and IL‐1β, exacerbating inflammaging (Liu et al. 2023).

A recent phase 2a randomized, placebo‐controlled clinical trial substantiated this hypothesis, demonstrating that low‐dose combinations of catalytic (BEZ235) and allosteric (RAD001) mTOR inhibitors in older adults were associated with a significant reduction in observed infection rates (Mannick et al. 2018). Multiple animal studies further corroborate these findings. For example, in aged mouse models, elevated mTOR activity strongly correlates with reduced antibody titers following influenza vaccination, whereas treatment with mTOR inhibitors (e.g., rapamycin) partially restores antibody responses and T‐cell function, suggesting that mTOR may be a key driver of vaccine efficacy decline (Montgomery and Shaw 2015). Additionally, interventions such as dietary restriction (e.g., calorie restriction) or autophagy activators (e.g., metformin) may indirectly enhance immune function by modulating mTOR activity (Martin‐Montalvo et al. 2013).

While these results highlight the therapeutic potential of mTOR modulation, the translation from animal models to enhanced vaccine efficacy in humans requires further confirmation in larger randomized controlled trials. Future research should focus on elucidating the dynamic regulatory mechanisms of mTOR signaling, exploring its cell‐specific effects across immune cell subsets, and leveraging multi‐omics approaches to uncover its interactions with aging‐related inflammatory networks. In summary, the mTOR pathway emerges as a critical nexus in immunosenescence and vaccine responsiveness, offering a robust theoretical foundation and promising research direction for developing immune‐enhancing strategies tailored to older populations.

## 3 How Aging Alters the Gut Microbiota

### 3.1 Age‐Related Microbial Dysbiosis and Gut Barrier Decline

Aging profoundly impacts the composition and functionality of the gut microbiota, with one of the most prominent features being a decline in microbial diversity, a change widely observed in older populations and closely tied to health status. Multiple studies indicate that with advancing age, the richness and evenness of the gut microbial community progressively diminish (Dong et al. 2023). Metagenomic analyses reveal that individuals over 80 years of age exhibit approximately 30% lower gut microbial α‐diversity compared to those under 40, a reduction strongly associated with the incidence of age‐related conditions such as cardiovascular disease and metabolic syndrome (Ghosh et al. 2022; Pang et al. 2023). Specifically, the abundance of beneficial bacteria, such as *Bifidobacterium* and *Lactobacillus*, is significantly reduced in older adults (Araujo et al. 2024; Ikeda et al. 2022; Sadoughi et al. 2022). These taxa play a vital role in maintaining gut homeostasis by metabolizing dietary fiber into short‐chain fatty acids (SCFAs), such as butyrate and propionate, which provide energy to intestinal epithelial cells and suppress inflammation (Muralitharan et al. 2025). Concurrently, the abundance of potentially pathogenic bacteria increases, including certain species within the *Clostridium* genus (e.g., *Clostridium difficile* ) and opportunistic pathogens in the *Proteobacteria* phylum (e.g., *Escherichia coli* ) (Parker et al. 2022). A U.S. cohort study of older adults found that *C. difficile* detection rates were three times higher in individuals over 65 compared to younger counterparts, exemplifying a characteristic dysbiotic pattern of “reduced beneficial bacteria and increased pathogens” in age‐related microbial dysbiosis (Dong et al. 2023). The underlying causes are multifaceted, likely involving dietary shifts (e.g., reduced fiber intake), slowed gut motility, antibiotic overuse, and immune decline (Larson et al. 2022).

### 3.2 Regulatory Mechanisms of the Gut‐Immune Axis

The gut‐immune axis represents a core mechanism of bidirectional interaction between the gut microbiota and the host immune system, modulating local and systemic immune homeostasis through metabolites, neural signals, and immune cell crosstalk. SCFAs, the primary metabolites of microbial fermentation of dietary fiber, are pivotal in this axis. These include acetate, propionate, and butyrate, with butyrate garnering particular attention for its pronounced roles in gut barrier protection and immune regulation. Research shows that butyrate enhances mucus secretion and the expression of tight junction proteins (e.g., zonula occludens‐1 [ZO‐1] and occludin) by providing energy to colonic epithelial cells (Dicks 2024). In vitro studies demonstrate that butyrate treatment increases ZO‐1 expression in human intestinal epithelial cells by approximately 60% and reduces barrier permeability by nearly 40%, effectively limiting the translocation of bacteria and their metabolites (e.g., lipopolysaccharide [LPS]) into the circulation and mitigating systemic inflammation risk (Tan et al. 2023). Additionally, SCFAs directly influence immune cell function by binding G protein‐coupled receptors (e.g., GPR41, GPR43, and GPR109A) or inhibiting histone deacetylases (HDACs) (Docampo et al. 2022). Butyrate promotes the differentiation of regulatory T cells (Tregs), increasing their proportion by about 50%, while enhancing anti‐inflammatory cytokine secretion (e.g., IL‐10) and suppressing the overactivation of pro‐inflammatory Th1 and Th17 cells, fostering immune tolerance and an anti‐inflammatory balance in the gut (Montgomery et al. 2024; Cao et al. 2024). This regulatory effect extends beyond the gut, influencing systemic immunity via peripheral circulation. Propionate and butyrate can enter the bloodstream, modulating hematopoietic stem cell differentiation in the bone marrow or enhancing the phagocytic capacity of alveolar macrophages to bolster anti‐infection defenses, highlighting the cross‐organ effects of the gut‐immune axis (Le Guern et al. 2023; Rowland et al. 2025).

However, the functionality of the gut‐immune axis heavily relies on microbial diversity and stability. When aging induces microbial dysbiosis, this regulatory capacity is markedly impaired. Studies indicate that the reduction in *Bifidobacterium* and increase in pathogenic bacteria in older adults directly correlate with diminished SCFA production (Ghosh et al. 2022; Bradley and Haran 2024). Simultaneously, dysbiotic microbiota may produce more pro‐inflammatory metabolites (e.g., secondary bile acids or aryl hydrocarbon receptor [AhR] antagonists), further activating TLR signaling and exacerbating inflammaging (Ziaka and Exadaktylos 2024). In models of chronic inflammatory bowel disease (IBD), reduced microbial diversity is strongly associated with Th17/Treg imbalance and approximately 50% higher levels of IL‐6 and TNF‐α (Yu et al. 2024). Moreover, the gut‐immune axis involves neuroendocrine pathways and synergistic interactions with gut‐associated lymphoid tissue (GALT) (Andrusaite et al. 2024). SCFAs indirectly modulate immune responses by activating the vagus nerve or regulating gut hormones (e.g., GLP‐1), while dendritic cells and M cells in GALT sample microbial antigens to initiate adaptive immune responses (Malik et al. 2023). In aged mice, antigen presentation efficiency of dendritic cells in GALT is approximately 30% lower than in young mice, resulting in inadequate T‐cell activation (Meng et al. 2023). This dysregulation weakens vaccine‐induced T‐ and B‐cell responses, providing a mechanistic basis for reduced vaccine efficacy. Thus, the gut‐immune axis, through SCFA‐mediated barrier protection and immune modulation, alongside dysbiosis‐driven immunosuppression, forms a complex dynamic equilibrium, offering a theoretical framework for understanding how aging impacts vaccine responses via microbial dysbiosis.

### 3.3 Vaccine Immunity and the Gut Microbiota

Mounting evidence underscores the critical role of the gut microbiota in vaccine‐induced immune responses, with alterations in its composition and function directly influencing vaccine effectiveness. In germ‐free or antibiotic‐treated mice with depleted gut microbiota, immune responses post‐vaccination are significantly diminished (Peng et al. 2009). Clinical studies reveal that an elevated Firmicutes/Bacteroidetes ratio correlates with increased antibody levels following BBIBP‐CorV vaccination (Tang et al. 2022), whereas reduced microbial diversity may lead to a 60% decrease in antibody titers (Huang et al. 2023). Probiotics (e.g., *Lactobacillus*) enhance vaccine‐induced secretory IgA (sIgA) levels by 2–3‐fold by promoting B‐cell activation in Peyer's patches (PPs), highlighting the indispensable role of the microbiota in initiating and sustaining adaptive immunity (Zhang et al. 2025). SCFAs, particularly butyrate, enhance B‐cell antibody secretion via HDAC inhibition, and exogenous SCFA supplementation can restore IgG titers by approximately 30% (Hwang et al. 2023). Additionally, tryptophan metabolites regulate interferon secretion by plasmacytoid dendritic cells (pDCs) via the AhR pathway, influencing vaccine cellular immunity (van der Sluis et al. 2025).

Conversely, probiotic supplementation experiments offer promising evidence for improving vaccine responses. In dysbiotic mice, supplementation with specific probiotics (e.g., *Bifidobacterium* or *Lactobacillus*) partially restores vaccine immunity (Mojgani et al. 2025). A study in aged mice demonstrated that pre‐vaccination oral administration of *Lactobacillus casei* increased IgG antibody titers by approximately 45%, enhanced T‐cell proliferation by about 35%, and reduced post‐vaccination inflammatory markers (e.g., IL‐6) by around 40% following influenza vaccination (Liu et al. 2024). Probiotic treatment increases the expression of the co‐stimulatory molecule CD80 on dendritic cells in GALT by 30%, optimizes the CD4^+^/CD8^+^ T‐cell ratio, and boosts T‐cell proliferation by 25% (Sivasankar et al. 2023; Marcinek et al. 2023). Certain strains also modulate gut Th17 and splenic Th1/Th2 ratios via the STING pathway (Pu et al. 2024). These effects may be linked to probiotics enhancing dendritic cell maturation, co‐activation of MHC class I/II molecules, and long‐term T‐cell activation through co‐stimulatory pathways like OX40‐OX40L (Mishra et al. 2023; Schettini et al. 2025). Probiotics correct age‐related Th2 skewing, increasing IFN‐γ (a Th1 marker) and decreasing IL‐4 (a Th2 marker), restoring the IgG2a/IgG1 ratio (Sivasankar et al. 2023).

Clinical relevance has been preliminarily validated in human studies. Multiple cohort studies suggest that gut microbiota composition in older adults is closely tied to COVID‐19 vaccine responses, with emerging nuances observed across different vaccine platforms. A prospective observational study from the University of Hong Kong found that older adults with higher neutralizing antibody levels post‐CoronaVac vaccination exhibited significantly greater baseline abundance of specific taxa (Peng et al. 2023). *Bifidobacterium* may indirectly enhance vaccine responses by modulating dendritic cell activation or boosting Th1 immunity (Qiu et al. 2024). A Korean cohort study showed that *Faecalibacterium prausnitzii* abundance positively correlates with antibody persistence following the BNT162b2 mRNA vaccine (Gao et al. 2023). The anti‐inflammatory properties of *F. prausnitzii* (e.g., butyrate production) may sustain immune memory by maintaining gut barrier integrity or regulating Treg function, while its reduction in older adults may accelerate antibody decline (Wang et al. 2022) (Kim et al. 2022). Additionally, a study from BC Children's Hospital in Canada found that individuals on high‐fiber diets (associated with increased SCFA‐producing bacteria) exhibited stronger COVID‐19 vaccine antibody responses, underscoring the influence of diet‐microbiota interactions on vaccine efficacy in older adults (Tang et al. 2022). Collectively, these findings indicate that age‐related microbial dysbiosis (e.g., reduced beneficial bacteria and abnormal diversity) is linked to diminished COVID‐19 vaccine responses, suggesting that microbial dysbiosis may be a key driver of age‐related vaccine efficacy decline.

However, most of these human studies are cross‐sectional or observational in nature and cannot establish causality. Intervention studies, particularly those using probiotics, provide more direct evidence. A recent systematic review and meta‐analysis of randomized controlled trials evaluated the effect of oral probiotics on vaccine responses in older adults (aged > 60 years) (Arioz Tunc et al. 2024). The review included ten RCTs involving 1560 participants and found that probiotics (particularly lactobacilli strains) may modestly enhance vaccine immunogenicity. Specifically, the average response ratios for antibody titres, seroprotection rate, and seroconversion rate were 1.3, 1.41, and 1.92, respectively, with seroconversion rate showing the most promising improvement (nearly twice that of placebo). Nevertheless, substantial heterogeneity was observed across probiotic strains, doses, vaccine types, and outcome measures, and several studies carried a high risk of bias due to missing outcome data.

### 3.4 Interactions Between mTOR and the Gut Microbiota

Metabolites of the gut microbiota, particularly SCFAs (e.g., butyrate and propionate), play a crucial role in modulating mTOR activity. Metabolites of the gut microbiota, particularly SCFAs (e.g., butyrate and propionate), play a crucial role in modulating mTOR activity. Metabolites of the gut microbiota, particularly short‐chain fatty acids (SCFAs) such as butyrate and propionate, have been shown in preclinical studies (primarily animal models and in vitro systems) to modulate mTOR activity. For example, butyrate, acting as an HDAC inhibitor, suppresses mTORC1 phosphorylation at S6K1 and 4EBP1 sites in cell culture and rodent models, thereby relieving its inhibition of autophagy and promoting intestinal epithelial cell repair (Dicks 2024). This effect was observed in maternal microbiota studies during pregnancy, where SCFAs modulated the transcriptomic profile of offspring stem cells via the mTOR pathway; blocking mTOR signaling abolished these microbiota‐mediated effects (Dang et al. 2025). Additionally, SCFAs activate GPR43/GPR109A receptors, upregulating AMP‐activated protein kinase (AMPK) activity, which antagonistically inhibits mTORC1 due to their opposing roles in energy sensing (Muralitharan et al. 2025; Docampo et al. 2022). These mechanisms have been demonstrated in cardiovascular disease models to improve cardiomyocyte metabolism (Pham et al. 2024). Moreover, SCFAs enhance tight junction protein expression (e.g., occludin) and suppress the NF‐κB pathway, reducing pro‐inflammatory cytokines such as IL‐6 and TNF‐α (Mann et al. 2024). Research shows that low SCFA levels in patients with *C. difficile* infection (CDI) correlate with barrier damage, and fecal microbiota transplantation improves outcomes by restoring SCFAs (Le Guern et al. 2023).

It should be noted that while these SCFA‐mTOR regulatory mechanisms are well‐supported in preclinical settings, direct causal evidence in human aging populations remains largely correlative. Human data are currently insufficient to establish definitive causality between SCFA‐mediated mTOR modulation and improved vaccine responses.

**FIGURE 3:**
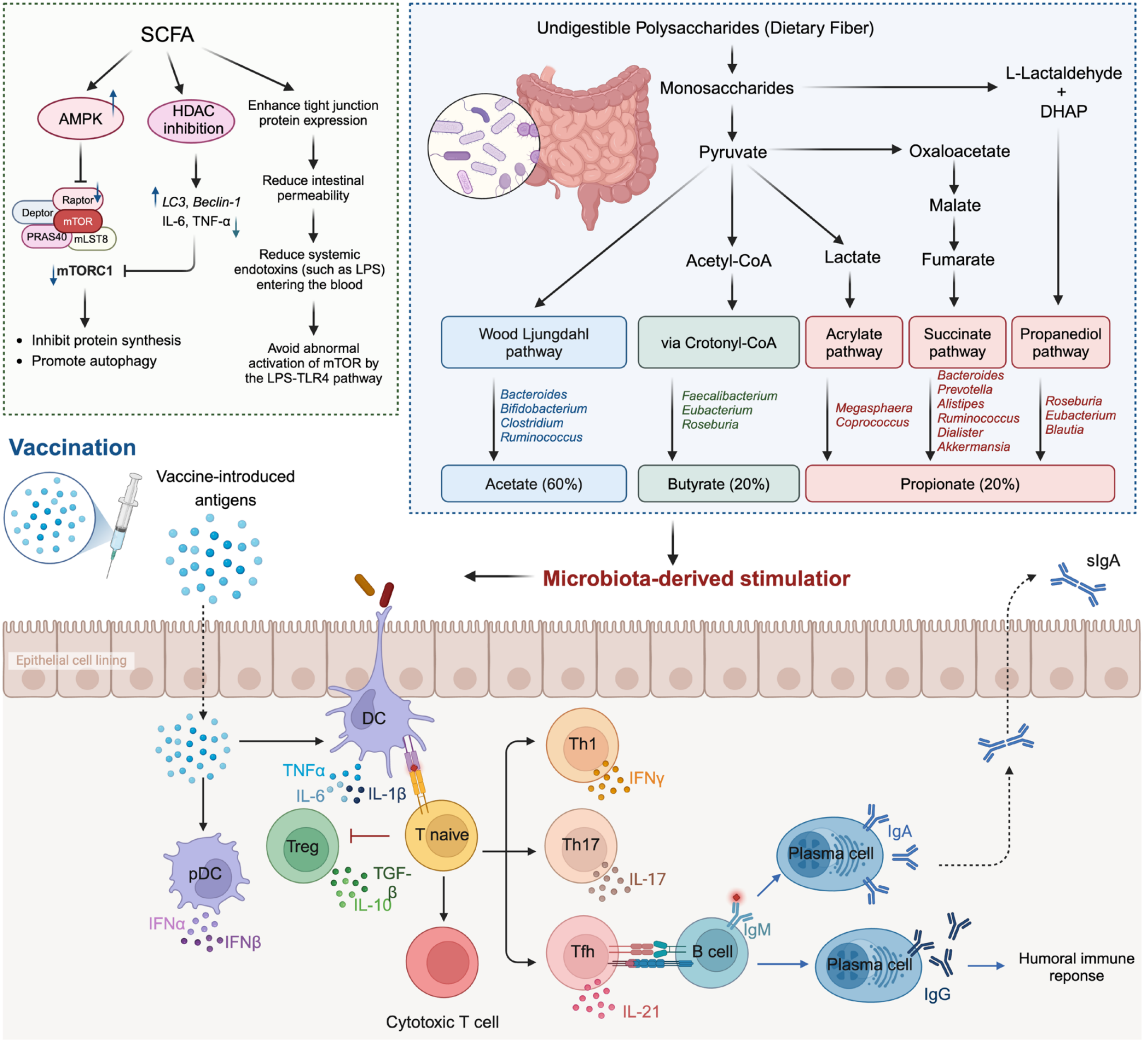
Regulation of the gut‐immune axis by short‐chain fatty acids (SCFAs) and its impact on vaccine responses. Dietary fiber fermentation by gut microbiota (e.g., *Bacteroides*, *Bifidobacterium*) produces SCFAs (acetate, butyrate, propionate), which inhibit mTORC1 via AMP‐activated protein kinase (AMPK) and histone deacetylase (HDAC) pathways, enhancing tight junction protein expression (e.g., ZO‐1, occludin) and reducing intestinal permeability. SCFAs promote autophagy, reduce inflammation, and support immune cell differentiation (e.g., Tregs, Th1, Th17), enhancing vaccine‐induced humoral immunity (IgG, IgA, sIgA). Dysbiosis in aging reduces SCFA production, impairing these protective mechanisms and vaccine efficacy.

The dynamic interplay between mTOR and the gut microbiota offers new perspectives for age‐related interventions. Current evidence suggests that combining mTOR inhibitors (e.g., rapamycin) with probiotics or high‐fiber diets may synergistically mitigate age‐related immune decline by restoring autophagy, boosting SCFA production, and enhancing gut barrier function. However, mTOR's dual role—supporting cellular growth while driving aging pathology—necessitates precise regulation to avoid adverse effects from excessive inhibition (Figure 3).

## 4 The Dynamic Network of the mTOR‐Microbiota‐Immune Axis in Aging

### 4.1 Immune Regulatory Mechanisms of the mTOR Signaling Pathway and Its Impact on Vaccine Efficacy

The mechanistic target of rapamycin (mTOR) signaling pathway exerts multifaceted, context‐dependent regulatory effects within the immune system. As a central integrator of metabolic cues and environmental signals, mTOR coordinates T‐cell differentiation, B‐cell antibody production, and innate immune responses, playing a dynamic and stage‐specific role in vaccine‐induced immunity (Wedemeyer et al. 2025; Pollizzi et al. 2015). mTOR operates through two distinct complexes—mTORC1 and mTORC2—with differential functions across immune cell subsets and response phases. During the early priming phase of vaccination, transient mTOR activation, particularly mTORC1, is essential for effective immune initiation. mTORC1 activates downstream targets such as S6 kinase 1 (S6K1) and 4E‐BP1, promoting the differentiation of naïve T cells into effector subsets (e.g., Th1 and Th17) and supporting rapid clonal expansion and metabolic reprogramming (Rosenlehner et al. 2024; Huckestein et al. 2024). Moderate mTORC2 activity further supports T‐cell survival, migration, and early effector functions via Akt and PKC pathways (Zhou et al. 2023). Studies show that mTORC2 knockout mice exhibit a 30% reduction in CD8^+^ T‐cell survival and a 25% decrease in migration efficiency to infection sites, underscoring its necessity in acute immune responses (Li et al. 2019). Similarly, mTOR signaling facilitates germinal center (GC) formation and B‐cell proliferation, which are critical for initial antibody production (Cho et al. 2016; Kometani et al. 2013). Thus, appropriate mTOR activation during the priming phase is necessary for robust vaccine‐induced effector responses.

In contrast, sustained or excessive mTORC1 activity, particularly in the later phases of the immune response, can impair the transition to memory cells. Persistent mTORC1 hyperactivity inhibits autophagy, promotes metabolic exhaustion, and upregulates inhibitory receptors (e.g., PD‐1, LAG‐3), thereby reducing memory T‐ and B‐cell formation and compromising long‐term protective immunity (Mannick and Lamming 2023; Ham et al. 2025). This exhaustion compromises the long‐term protective capacity of vaccines; for instance, post‐influenza vaccination, memory T cells in aged mice are only one‐third as abundant as in young mice (Ham et al. 2025). In B cells, while mTOR supports antibody production through mRNA translation and metabolic reprogramming, excessive activation disrupts germinal center reactions and antibody affinity maturation (Alsoussi et al. 2023). Studies show that B cells overexpressing mTORC1 exhibit a 45% reduction in germinal center reaction efficiency and a 30% drop in IgG titers, resulting in suboptimal antibody quality post‐vaccination (Li et al. 2019). Furthermore, mTOR exhibits a double‐edged effect in innate immunity. It enhances antigen presentation by macrophages and dendritic cells—for example, mTORC1 activation increases CD80 expression on dendritic cells by approximately 35%, facilitating initial vaccine antigen recognition (Qi et al. 2024). However, prolonged activation induces pro‐inflammatory (M1) polarization, elevating IL‐6 and TNF‐α secretion by about 50%, exacerbating inflammaging and suppressing adaptive immune initiation (Zhang et al. 2023). In vitro co‐culture experiments demonstrate that M1 macrophages reduce T‐cell activation efficiency by approximately (De Biasi et al. 2024).

This dual effect manifests as a dynamic balance in vaccine responses. Moderate, transient mTOR activation facilitates rapid establishment of antigen‐specific immunity, whereas uncontrolled or sustained hyperactivity impairs immune memory and protective efficacy. Mouse studies show that early administration of the mTOR inhibitor rapamycin reduces antibody responses by approximately 30%, but its use in later immune phases enhances memory T‐cell generation by about 45% via autophagy promotion (Withers et al. 2025). A study on pneumococcal vaccination found that rapamycin‐treated groups exhibited a 50% increase in memory T‐cell proportions and a twofold extension of protection duration (Sagawa et al. 2023). Moreover, clinical trials (NCT04420338 and NCT04409327) exploring mTOR inhibitors (e.g., rapamycin) for improving vaccine efficacy report that pre‐vaccination rapamycin use increases antibody titers by 20% and reduces inhibitory CD4^+^ and CD8^+^ T‐cell populations, though indirect mTORC2 suppression may impair memory B‐cell function (Mannick et al. 2018, 2014, 2021; Withers et al. 2025).

**FIGURE 4:**
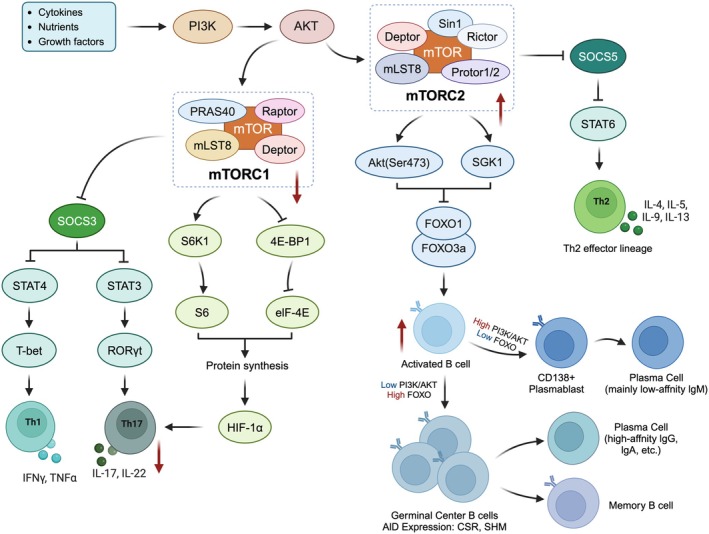
Metabolic regulation of immune cells by the mTOR pathway and its implications for vaccine responses. The mTOR pathway integrates metabolic cues (e.g., glucose, serine, ATP) to regulate immune cell metabolism via mTORC1 and mTORC2. mTORC1 drives glycolysis, lipid synthesis, and glutaminolysis, promoting Th1, Th17, and germinal center (GC) B‐cell differentiation, while mTORC2 supports oxidative phosphorylation (OXPHOS) and Th‐mediated B‐cell responses. Dysregulated mTOR in aging enhances glycolysis and impairs OXPHOS, reducing plasma cell differentiation and memory B‐cell formation, thus weakening vaccine responses. AMPK counteracts mTORC1, promoting fatty acid oxidation (FAO) and autophagy to support immune cell survival.

These findings provide population‐level evidence of mTOR's influence on vaccine immunity. In summary, a balanced, stage‐appropriate modulation of mTOR signaling—rather than broad inhibition—represents a promising strategy to optimize vaccine efficacy in aging populations (Figure 4).

### 4.2 Dynamic Interactions of the mTOR‐Microbiota‐Immune Axis in Aging

mTOR activity markedly increases with age, profoundly impacting immune function deterioration and the health of older individuals. mTOR activity markedly increases with age, profoundly impacting immune function deterioration and the health of older individuals (Liu et al. 2022). Preclinical evidence from aged mouse models indicates that elevated mTOR activity accelerates immunosenescence by suppressing autophagy, leading to mitochondrial dysfunction and elevated ROS levels in T cells (Panwar et al. 2023; Sohel 2025). It also promotes pro‐inflammatory cytokine secretion (e.g., IL‐6 and TNF‐α) via NF‐κB signaling, thereby intensifying inflammaging (Mannick and Lamming 2023; Ba et al. 2023). This inflammatory microenvironment not only hastens immune cell aging but also amplifies systemic inflammation through positive feedback, forming a vicious cycle that further weakens immune responsiveness (Zhao et al. 2024).

In the intestine, animal studies show that elevated mTOR activity in epithelial cells impairs autophagy, resulting in reduced barrier integrity and increased permeability. In aged mice (24 months), mTOR inhibitors such as rapamycin restore autophagy and reduce gut permeability (Telpaz and Bel 2023; Hu et al. 2023). In aged mice (24 months), epithelial expression of autophagy genes (*Atg5* and *LC3*) drops by about 50%, and mTORC1 phosphorylates the autophagy initiator ULK1, suppressing autophagosome formation, reducing epithelial autophagy, and hindering barrier repair, leading to “leaky gut” (Kazyken et al. 2024; Park et al. 2023). mTOR inhibitors (e.g., rapamycin) restore autophagy, increasing LC3‐II levels by approximately 60% and reducing gut permeability by about 40%, significantly ameliorating leakiness (Regan et al. 2022). Additionally, mTOR indirectly influences microbial ecology by altering cellular metabolic demands. mTORC1 activates S6K1, enhancing glycolysis gene transcription and shifting metabolism toward simple sugar utilization, reducing dietary fiber metabolism and inhibiting the growth of SCFA‐producing beneficial bacteria (e.g., *Bifidobacterium*), exacerbating dysbiosis (Panwar et al. 2023). Conversely, SCFAs (e.g., butyrate and propionate) counterregulate mTOR activity via AMPK and HDAC pathways (Tan et al. 2023). Butyrate, an HDAC inhibitor, suppresses HDAC3, promotes histone acetylation, and reduces mTORC1 expression (Xu and Wan 2023). SCFAs also activate AMPK via GPR43, phosphorylating TSC2 to inhibit mTORC1, forming an antagonistic effect (Docampo et al. 2022). This dual regulation enhances autophagy, repairs the gut barrier, and optimizes epithelial metabolic homeostasis via mTORC1 downstream 4E‐BP1 deactivation (Panwar et al. 2023).

**FIGURE 5:**
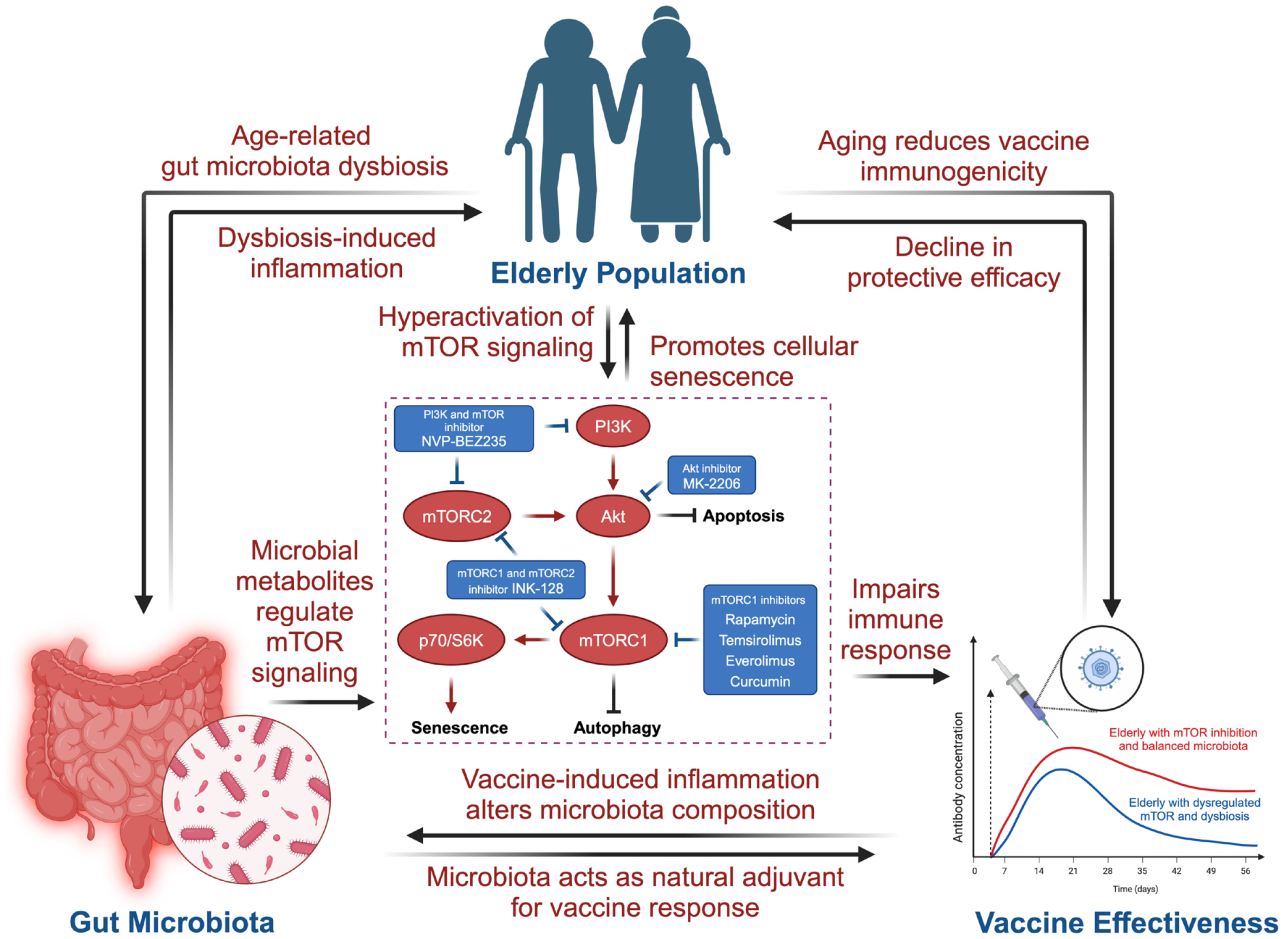
Impact of aging on vaccine immunogenicity through the mTOR‐microbiota‐immune axis. Aging induces gut microbiota dysbiosis, reducing microbial metabolites that regulate mTOR signaling, leading to mTOR hyperactivity, cellular senescence, and inflammation. This promotes immunosenescence, impairing immune responses and decreasing vaccine protective efficacy in older adults. mTORC1 inhibitors (e.g., rapamycin, temsirolimus, everolimus) and AKT inhibitors (e.g., MK‐2206) mitigate these effects by enhancing autophagy and reducing senescence, while microbial metabolites act as natural adjuvants to enhance vaccine responses. A graph illustrates the decline in antibody concentrations post‐vaccination in elderly individuals with dysregulated mTOR and microbiota compared to those with balanced profiles.

In immune regulation, SCFAs influence T‐cell differentiation via the AMPK‐mTOR axis (Trujillo‐Ochoa et al. 2023). High mTORC1 activity suppresses Foxp3 expression, hindering Treg development, whereas SCFA‐activated AMPK upregulates Foxp3 transcription, forming a complex with Runx1 to antagonize RORγt, inhibiting Th17 differentiation and shifting the Th17/Treg balance toward Tregs, with increased IL‐10 secretion, alleviating inflammaging (Mann et al. 2024; Ma et al. 2023; McGettigan et al. 2024). SCFAs also modulate B‐cell function via mTOR, reducing mTORC1 activity to enhance germinal center reactions and antibody class‐switching. In aging, excessive mTORC1 activation and microbial dysbiosis form a positive feedback loop: mTOR suppresses SCFA‐producing bacteria, and reduced SCFAs relieve mTOR inhibition, worsening immunosuppression (Ikeda et al. 2022; Tan et al. 2023; Rangan and Mondino 2022). Studies show that rapamycin upregulates autophagy by blocking the mTORC1‐S6K1 pathway, while probiotics restore SCFA production, synergistically improving barrier function and Treg activity, suggesting that AMPK/HDAC‐mediated SCFA effects are a critical bridge (Qin et al. 2024) (Figure 5).

## 5 Future Directions and Clinical Implications

With deepening insights into the mTOR pathway, gut microbiota, and their interactions with vaccine immunity, future research and clinical practice may benefit from multidisciplinary, precision‐medicine approaches. These could include combining mTOR‐targeted agents with microbiota‐modulating strategies to potentially enhance immune protection in older adults. Preclinical studies suggest that rapamycin can restore autophagy and reduce inflammation by inhibiting mTOR, while certain probiotics may increase SCFA production, strengthen gut barrier function, and support immune regulation.

We hypothesize that the appropriately timed combined use of mTOR inhibitors and microbiota modulation may improve antibody titers and T‐cell responses, based on evidence from aged mouse models. However, successful clinical translation demands careful consideration of several practical parameters, particularly potential differences across vaccine platforms.

Different vaccine technologies—including mRNA‐based, inactivated, protein subunit, viral vector, and adjuvanted vaccines—engage innate sensing pathways, germinal center dynamics, and metabolic reprogramming to varying degrees. Consequently, microbiota‐derived metabolites and mTOR signaling may exert differential modulatory effects depending on the vaccine platform. For example, mRNA vaccines strongly activate type I interferon responses and glycolytic reprogramming, processes that are highly sensitive to mTOR activity. In contrast, inactivated or adjuvanted protein subunit vaccines may depend more heavily on adjuvant‐driven antigen presentation and could interact differently with SCFA‐mediated immune regulation and gut barrier function. Emerging evidence also indicates platform‐specific changes in gut microbiota composition following vaccination, as well as differential associations between specific microbial taxa and immunogenicity (Mannick et al. 2018, 2014, 2021).

Translating these insights requires a structured clinical framework. First, regarding intervention timing, mTOR inhibition may be most beneficial when administered shortly before or in the post‐priming phase rather than during peak effector responses. Second, for dosing strategies, regimens should favor low‐dose, intermittent, or short‐course approaches. Third, addressing safety profiles in older adults with multimorbidity and polypharmacy requires particular attention. mTOR inhibitors carry risks of immunosuppression, hyperglycemia, hyperlipidemia, mucositis, and increased infection susceptibility, which may be amplified in frail individuals or those receiving multiple medications. Potential drug–drug interactions and additive immunosuppressive effects must be rigorously evaluated. Although probiotics and dietary interventions generally exhibit favorable safety profiles, risks such as gastrointestinal intolerance or bacterial translocation in severely immunocompromised patients cannot be overlooked. Within this framework, any future strategy should incorporate individualized risk–benefit assessments and structured clinical monitoring parameters, including regular evaluation of infection rates, metabolic markers, inflammatory indices, and vaccine‐specific correlates.

Multi‐omics technologies will serve as powerful tools for elucidating the molecular details of the mTOR‐microbiota‐immune axis and for identifying likely responders. Future studies should prioritize well‐designed, randomized controlled trials that stratify participants by age, frailty status, comorbidities, baseline microbiota composition, and mTOR activity. These trials should systematically evaluate combined interventions across vaccine platforms (e.g., mRNA and inactivated vaccines) and stages of aging, while incorporating clear feasibility endpoints such as adherence, adverse event rates, and cost‐effectiveness.

Current “one‐size‐fits‐all” vaccination approaches often overlook individual differences in microbiota composition and mTOR activity, which may be important determinants of low responsiveness. Observational studies have reported that older adults with higher microbial diversity or SCFA levels tend to exhibit stronger antibody responses to influenza vaccines, suggesting that microbiota features could serve as potential predictive markers of vaccine efficacy. Clinically, non‐invasive assessments could help inform personalized vaccination strategies. For example, pre‐vaccination microbiota optimization via high‐fiber diets or targeted probiotics, or selective use of low‐dose mTOR inhibitors in identified poor responders may enhance immune responses. The development of novel vaccine adjuvants (e.g., SCFA analogs) or microbiota‐informed vaccine platforms also represents a promising direction.

Nevertheless, these approaches face substantial challenges, including standardization of interventions, validation of long‐term efficacy and safety, and evaluation of cost‐effectiveness and health equity. Future trials must address the marked heterogeneity of older populations and incorporate large‐scale, multicenter designs to confirm generalizability and feasibility. Ethical concerns—particularly the potential risks of immune modulation in vulnerable elderly individuals—must be carefully weighed. Ultimately, a nuanced integration of mTOR‐targeted therapies, microbiota modulation, and multi‐omics‐guided personalization may not only help clarify the mechanisms underlying diminished vaccine responses in aging but also advance actionable precision medicine strategies for immune protection in older adults, addressing infectious disease challenges in aging societies more effectively.

## 6 Conclusion

Aging significantly influences vaccine efficacy by altering the gut microbiota and mTOR pathway, a process involving reduced microbial diversity, decreased SCFA production, and mTOR hyperactivity‐driven inflammaging and immune decline. While preclinical models robustly demonstrate these interactions, we emphasize that direct causal evidence in human aging remains an important frontier requiring validation. As a bridge linking microbial metabolites to immune responses, mTOR regulates T‐cell differentiation and antibody production while dynamically interacting with SCFAs and other metabolites, affecting vaccine‐induced protection and providing novel insights into this complex network in aging. Existing studies, through animal models and preliminary human data, have validated the potential of mTOR inhibitors and probiotics to enhance vaccine efficacy, highlighting their clinical promise. However, translating these findings into practice requires integrating mechanistic research with intervention trials to systematically elucidate the molecular basis and individual variability of the mTOR‐microbiota‐immune axis. Accordingly, developing personalized vaccine strategies for older adults—such as combining microbiota profiling with precision interventions—will be a critical pathway to address public health challenges in aging societies. This approach not only promises to reduce the infectious disease burden in older populations but also opens new avenues for research and application in healthy aging and precision medicine.

## Author Contributions

**Jiaxuan Li:** writing – original draft and writing – review and editing. **Yuhong Zhang:** writing – original draft. **Daijun Yu:** writing – original draft. **Jianhua Li:** project administration. **Keda Chen:** project administration. **Lisheng Chu:** writing – review and editing.

## Funding

This study was funded by the Provincial Industry‐University Cooperation Collaborative Education Project (No. 318 [2022] of the Zhejiang Development Reform Society) and the University Level Scientific Research Project of Zhejiang Shuren University (Grant No. 2024R057).

## Ethics Statement

The authors have nothing to report.

## Consent

The authors have nothing to report.

## Conflicts of Interest

The authors declare no conflicts of interest.

## Data Availability

Data sharing not applicable to this article as no datasets were generated or analysed during the current study.
